# A study of risk factors for tuberculous meningitis among patients with tuberculosis in China: An analysis of data between 2012 and 2019

**DOI:** 10.3389/fpubh.2022.1040071

**Published:** 2023-01-27

**Authors:** Mailing Huang, Yan Ma, Xinyu Ji, Hui Jiang, Fangchao Liu, Naihui Chu, Qi Li

**Affiliations:** ^1^Department of Tuberculosis, Beijing Chest Hospital, Capital Medical University, Beijing, China; ^2^Institute of Basic Research in Clinical Medicine, China Academy of Chinese Medical Sciences, Beijing, China; ^3^Division of Science and Technology, Beijing Chest Hospital, Capital Medical University, Beijing, China; ^4^Clinical Center on Tuberculosis, Beijing Chest Hospital, Capital Medical University, Beijing, China

**Keywords:** tuberculosis, tuberculous meningitis, miliary pulmonary tuberculosis, risk factors, malnutrition

## Abstract

**Purpose:**

The present study aimed to explore the risk factors for tuberculous meningitis (TBM) among patients with tuberculosis (TB).

**Methods:**

This retrospective study was conducted on patients with TB who were hospitalized in Beijing Chest Hospital between January 2012 and December 2019. Demographic and clinical data of patients with TB were extracted from electronic medical records using a standardized data collection system. Logistic regression was used to analyze the risk factors associated with TBM.

**Results:**

Of the total number of 22,988 cases enrolled, 3.1% were cases of TBM, which included 127 definite and 581 probable TBM, respectively. Multivariate analysis showed that definite TBM was significantly associated with patients aged < 30 years [adjusted odds ratio (aOR) = 3.015, 95% confidence interval (CI): (1.451–6.266)], who were farmers [aOR = 1.490, 95%CI: (1.020–2.177)], with miliary pulmonary TB [aOR = 105.842, 95%CI: (71.704–156.235)], and with malnutrition [aOR = 2.466, 95%CI: (1.110–5.479)]. Additionally, probable TBM was significantly associated with patients aged < 30 years [aOR = 2.174, 95% CI: (1.450–3.261)], aged 30–59 years [aOR = 1.670, 95% CI: (1.222–2.282)], who were farmers [aOR = 1.482, 95%CI: (1.203–1.825)], with miliary pulmonary TB [aOR = 108.696, 95%CI: (87.122–135.613)], and with a digestive system TB [aOR = 2.906, 95%CI: (1.762–4.793)].

**Conclusion:**

An age of < 30 years, being a farmer, and having miliary pulmonary TB were risk factors for TBM among patients with TB. Further screening of patients with TB with aforementioned characteristics could facilitate clinicians to identify patients with TBM at an early stage.

## Introduction

Tuberculosis (TB) is ranked as the second leading cause of death from a single infectious agent after coronavirus 2019 (COVID-19) (1), and tuberculous meningitis (TBM) is the most severe form of TB (2). Despite 1-5% of TBM among cases of TB, it is universally fatal if left untreated (3, 4). Previous studies demonstrated that TBM had high mortality and morbidity, and the death rate reached up to 20-30% in TBM without HIV (human immunodeficiency virus) infection, but up to 50-60% in TBM with HIV infection (5–9). Moreover, nearly half of the survivors would have neurological sequelae, even if they are receiving treatment.

An early diagnosis of TBM can facilitate timely treatment and improve prognosis ultimately. However, it is difficult to diagnose TBM. Its symptoms and signs are variable and nonspecific. Existing diagnostic technologies still have some limitations. Elevated protein, lymphocytic pleocytosis, and low glucose with cerebrospinal fluid (CSF) cannot reliably distinguish TBM from other subacute meningitis types (cryptococcal meningitis, etc.) (10). Neuroimaging, especially cerebral magnetic resonance imaging (MRI), which is sensitive to the detection of the lesion, exhibits low specificity (11). Microbiological tests have good specificity but unfavorable sensitivity. Adenosine deaminase and T-SPOT.TB testing have different sensitivities and specificities and are expensive (12, 13). Notably, most of the aforementioned tests are unavailable in a resource-limited area. Risk factors for TBM should be identified, which could help clinicians to facilitate an early detection of TBM, especially in medical resource-limited areas.

Literature published demonstrated that being a farmer (14), having diabetes mellitus (15), having kidney failure (15–17), having HIV infection (1), having malnutrition (1), and having a rheumatic disease (18) are the risk factors for TB. Patients with the aforementioned characteristics are more likely to have TB, which can help clinicians identify TB early. Previous studies reported that TBM was the second stage of TB (19, 20), which was accompanied by the occurrence of any other TB. Up to 80% of miliary pulmonary TB is accompanied by TBM, and TBM was especially common in young children and people with untreated HIV infection (21, 22).

Early detection of TBM and targeted interventions are crucial for reducing the risk of TBM.

Thus, this study aimed to determine different factors associated with TBM in China.

## Methods

### Study population and area

Patients with TB who were hospitalized at Beijing Chest Hospital from 1 January 2012 to 31 December 2019 were enrolled in this retrospective study. Beijing Chest Hospital is a Class-3A level (the top level of hospital ranking in China) specialized hospital for treating TB infectious diseases in Beijing (north of China), equipped with 1,100 beds, and designated as a municipal-level hospital for treating patients with TB.

The participants were excluded, if they belonged to any of the following patient categories: (1) HIV-infected patients with TB; (2) possible cases of TBM according to the uniform case definition; and (3) suspected cases of TBM without results of cerebral imaging and lumbar puncture.

### Definitions

In the present study, patients were categorized as definite, probable, possible TBM, and non-TBM cases, based on a uniform case definition for use in clinical research on TBM (23). Briefly, three types of TBM, including definite, probable, and possible TBM of diagnostic criteria, are defined as follows: (1) definite TBM: microbiological identification or evidence from commercial nucleic acid amplification tests of the central nervous system (CNS) mycobacterium tuberculosis infection; (2) probable TBM: when imaging is available, a diagnostic score of 12 or above is required, and when imaging is not available, a diagnostic score of 10 or above is required; and (3) possible TBM: when imaging is available, a diagnostic score of 6–11 is required, and when imaging is not available, a diagnostic score of 6–9 is required.

The definition of miliary pulmonary TB included the presence of a miliary pattern on chest radiograph or computed tomography (CT), along with one or more of the following features: (1) clinical features compatible with pulmonary TB, including cough for a duration of 3 weeks or more, fever, weight loss, night sweats, loss of appetite, or hemoptysis; (2) microbiological and/or histopathological evidence of TB; and (3) response to antituberculosis treatment (24, 25).

Secondary pulmonary tuberculosis, also known as reactivated or adult pulmonary tuberculosis, refers to the reactivation of dormant tuberculosis lesions or re-exogenous infection.

Malnutrition was defined as the body mass index of patients (BMI) of < 18.5 kg/m^2^ according to the consensus statement of the European Society for Clinical Nutrition and Metabolism (ESPEN) (26). The BMI was calculated as weight in kilograms (kg) divided by height in meter square (kg/m^2^).

In this study, patients with TB included secondary pulmonary TB, miliary pulmonary TB, tuberculous pleuritis, tuberculous peritonitis, tuberculous pericarditis, lymph node TB, cutaneous and soft tissue TB, TB of the head and the neck, digestive system TB, genitourinary TB, vertebral TB, and osteoarticular TB, as seen in Table 1.

**Table 1:** Demographic and clinical characteristics of 22,988 cases from 2012 to 2019.

**Characteristics**	**Total (*n =* 22,988)**	**Non-TBM (*n =* 22,280)**	**Definite TBM (*n =* 127)**	**Probable TBM (*n =* 581)**	**P-value**
**Age group, years**					
< 30	6,358 (27.7)	6,024 (27.0)	68 (53.6)	266 (45.8)	**< 0.001**
30–59	9,694 (42.1)	9,441 (42.4)	38 (29.9)	215 (37.0)	
≥60	6,936 (30.2)	6,815 (30.6)	21 (16.5)	100 (17.2)	
**Sex**					**< 0.001**
Male	14,714 (64.0)	14,322 (64.3)	73 (57.5)	319 (54.9)	
Female	8,274 (36.0)	7,958 (35.7)	54 (42.5)	262 (45.1)	
**Marital status**					**< 0.001**
Married	15,288 (66.5)	14,916 (66.9)	64 (50.4)	308 (53.0)	
Unmarried	6,083 (26.5)	5,784 (26.0)	57 (44.9)	242 (41.7)	
Others	1,617 (7.0)	1,580 (7.1)	6 (4.7)	31 (5.3)	
**Occupation**					**< 0.001**
Farmer	7,552 (32.9)	7,242 (32.5)	55 (43.3)	255 (43.9)	
Other	15,436 (67.1)	15,038 (67.5)	72 (56.7)	326 (56.1)	
**Comorbidity**					
Diabetes mellitus	4,124 (17.9)	4,059 (18.2)	12 (9.4)	53 (9.1)	**< 0.001**
Hypertension	3,362 (14.6)	3,289 (14.8)	9 (7.1)	64 (11.0)	**0.002**
Coronary heart disease	1,392 (6.1)	1,376 (6.2)	2 (1.6)	14 (2.4)	**< 0.001**
Liver cirrosis	195 (0.8)	193 (0.9)	1 (0.8)	1 (0.2)	0.098
Kidney failure	525 (2.3)	506 (2.3)	6 (4.7)	13 (2.2)	0.182
Rheumatic disease	537 (2.3)	507 (2.3)	3 (2.4)	27 (4.6)	**0.001**
Carcinoma	1,188 (5.2)	1,183 (5.3)	2 (1.6)	3 (0.5)	**< 0.001**
Malnutrition	563 (2.4)	532 (2.4)	8 (6.3)	23 (4.0)	**0.001**
**Concurrent TB**					
Secondary pulmonary TB	15,847 (68.9)	15,349 (68.9)	100 (78.7)	398 (68.5)	0.056
Miliary pulmonary TB	650 (2.8)	249 (1.1)	68 (53.5)	333 (57.3)	**< 0.001**
Tuberculous pleuritis	4,993 (21.7)	4,872 (21.9)	21 (16.5)	100 (17.2)	**0.01**
Tuberculous peritonitis	324 (1.4)	308 (1.4)	2 (1.6)	14 (2.4)	0.115
Tuberculous precarditis	183 (0.8)	180 (0.8)	1 (0.8)	2 (0.3)	0.516
Lymph node TB	1,189 (5.2)	1,121 (5.0)	8 (6.3)	60 (10.3)	**< 0.001**
Cutaneous and soft tissue TB	1,170 (5.1)	1,142 (5.1)	5 (3.9)	23 (4.0)	0.378
TB of the head and neck	112 (0.5)	104 (0.5)	2 (1.6)	6 (1.0)	**0.023**
Digestive system TB	341 (1.5)	298 (1.3)	3 (2.4)	40 (6.9)	**< 0.001**
Genitourinary TB	289 (1.3)	259 (1.2)	4 (3.1)	26 (4.5)	**< 0.001**
Vertebral TB	2,032 (8.8)	1,927 (8.6)	11 (8.7)	94 (16.2)	**< 0.001**
Osteoarticular TB	1,336 (5.8)	1,299 (5.8)	4 (3.1)	33 (5.7)	0.432

Data are presented as n (%). TBM, tuberculous meningitis; TB, tuberculosis.

P values are compared from chi-square (χ^2^) test, Fisher's exact test, and Kruskal–Wallis test, as appropriate.

The value of *P* < 0.05 indicates statistical significance.

In the present study, TBM included definite TBM and probable TBM. Non-TBM cases included all other cases of TB without TBM.

### Data collection

The present study collected demographic (including age, sex, marital status, and occupation) and clinical (comorbidities and concurrent TB) data for all hospitalized patients with TB from electronic medical records. In the hospital, all diseases are coded using the International Classification of Diseases, 10th Revision with Clinical Modification (ICD-10-CM). The information about patients was collected only during the first hospitalization, but not if the patient was hospitalized repeatedly. Moreover, to reclassify patients with TBM into definite, probable, and possible TBM according to the uniform case definition, the present study also collected symptoms and symptom duration, signs, history of recent (within the past 1 year) close contact with an individual with pulmonary TB, routine biochemistry and biochemical analysis of the cerebrospinal fluid (CSF), imaging (computed tomography (CT), magnetic resonance imaging (MRI), or ultrasound (US)) of the cereba and other organs, mycobacterial evidence by any of the smear microscopy, culture, polymerase chain reaction (PCR), Xpert from sputum, urine, and stool, pleural effusion, pericardial effusion, peritoneal effusion, pus, and bronchoalveolar lavage fluid from patients with TBM.

### Statistical analysis

Categorical variables were described as frequency and percentages (%) and continuous variables as mean and standard deviation (SD) or median and interquartile range (IQR), as appropriate. The means for continuous variables were compared using the independent group variance analysis when the data were normally distributed; otherwise, the Kruskal–Wallis test was used. A comparison of categorical variables was done using the chi-square (χ^2^) test or the Fisher exact test if the cell counts were small. To investigate and identify the risk factors of TBM further, the present study generated multivariate logistic regression analyses. Odds ratios (ORs) and 95% confidence intervals (CIs) were calculated to demonstrate the risk for TBM. Statistical analysis was conducted using SPSS software (version 19.0, IBM, Armonk, NY, USA), and a value of P of < 0.05 indicated statistical significance.

### Ethics statement

The study was approved by the Ethics Committee of Beijing Chest Hospital (20210113YJS-2021-007). In the present study, the retrospective collection and analysis of cases were patients' demographic characteristics and diagnostic information. All data were supplied and analyzed, without access to personal identifiable information (PII). No informed consent was required.

## Results

### Demographics and clinical characteristics of patients

**Figure 1:**
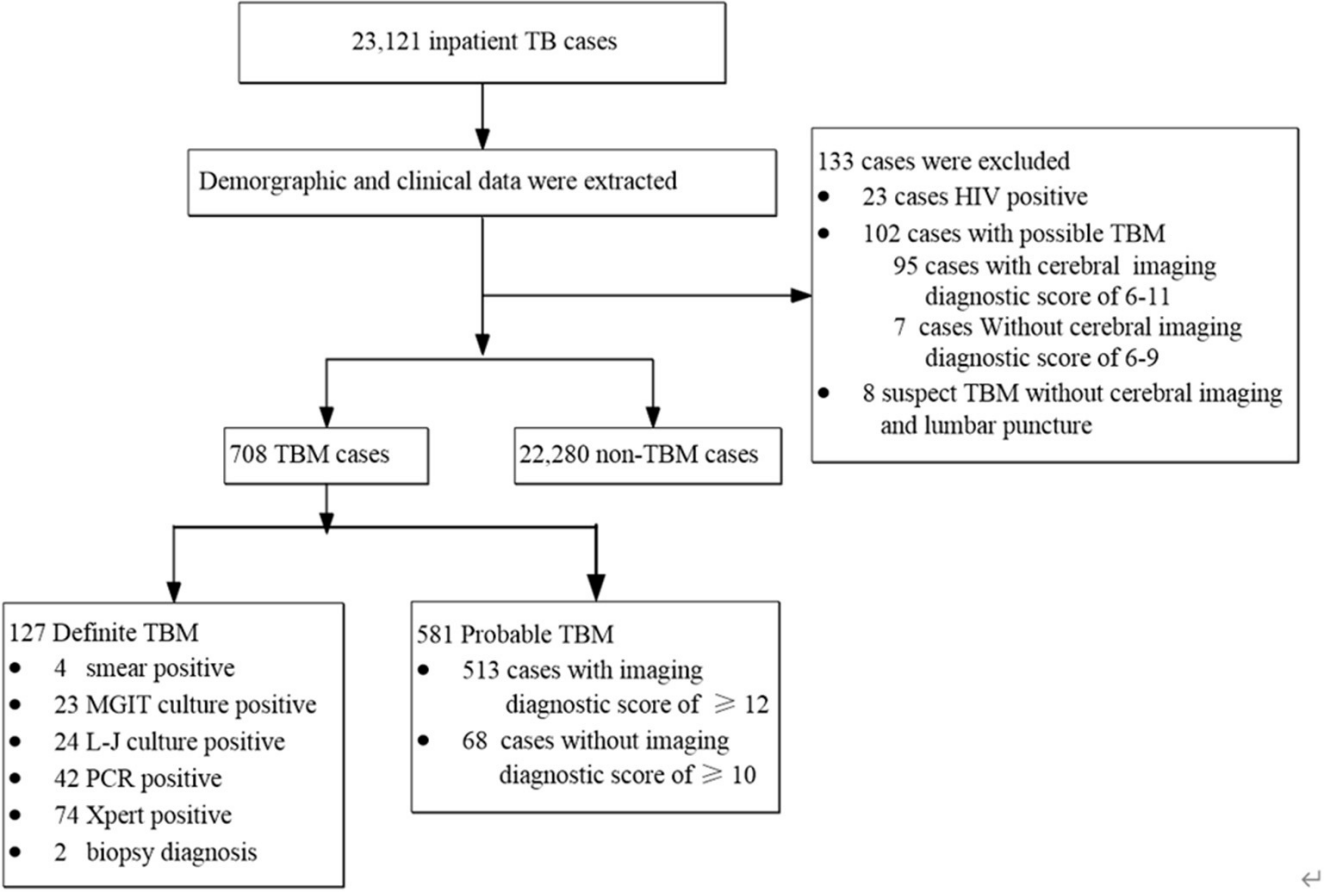
Flowchart of subjects enrolled in the study.

A total of 23,121 inpatient cases with TB were enrolled in this study between 2012 and 2019. Of these, 133 cases were excluded, including 102 possible cases of TBM diagnosed by the uniform case definition, 8 suspected cases of TBM who did not undergo lumbar puncture and cerebral imaging, and 23 HIV-positive cases. A total of 22,988 patients with TB were thus included in the present study (Figure 1).

The median age of the patient was 47 years, and 64.0% of patients were men. Of these, 17.9% and 14.6% had diabetes mellitus and hypertension, respectively. Of 22,988 patients, 708 (3.1%) cases were patients with TBM, including 127 definite and 581 probable cases, and 22,280 (96.9%) cases were patients without TBM. Patients with definite and probable TBM were more likely to be younger, and nearly two-thirds of patients without TBM were women. Patients with definite and probable TBM were more likely to be male, to be less than aged 30 years, and to have rheumatic disease and malnutrition than patients without TBM. They were also more likely to have miliary pulmonary TB. The percentage of definite patients with TBM categorized as having miliary pulmonary TB (53.5%) was more than 48-fold greater than the percentage of patients without TBM (1.1%). Moreover, the percentage of probable TBM patients having miliary pulmonary TB (57.3%) was more than 52-fold greater than the percentage of patients without TBM (Table 1).

**Figure 2:**
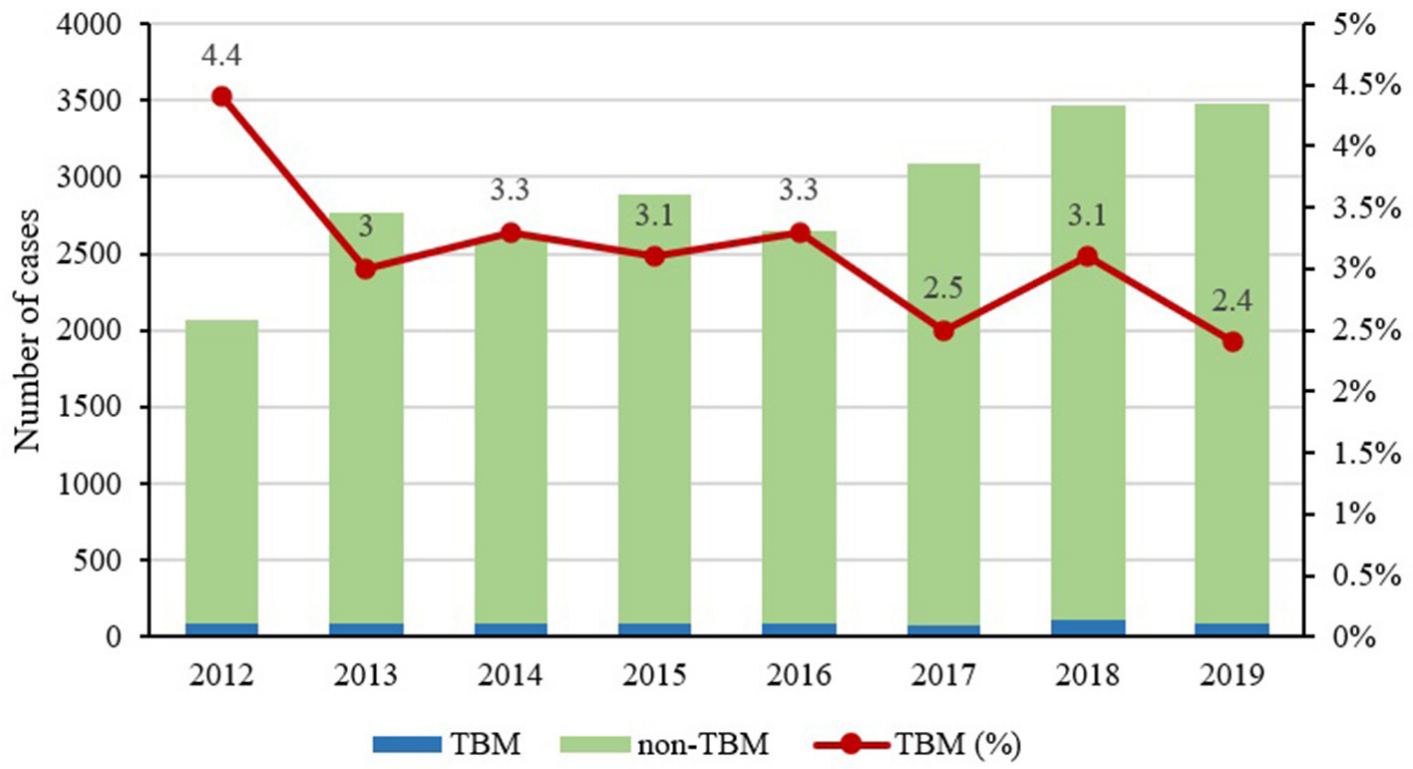
Proportion of tuberculosis (TB) and tuberculous meningitis (TBM) from 2012 to 2019.

**Figure 3:**
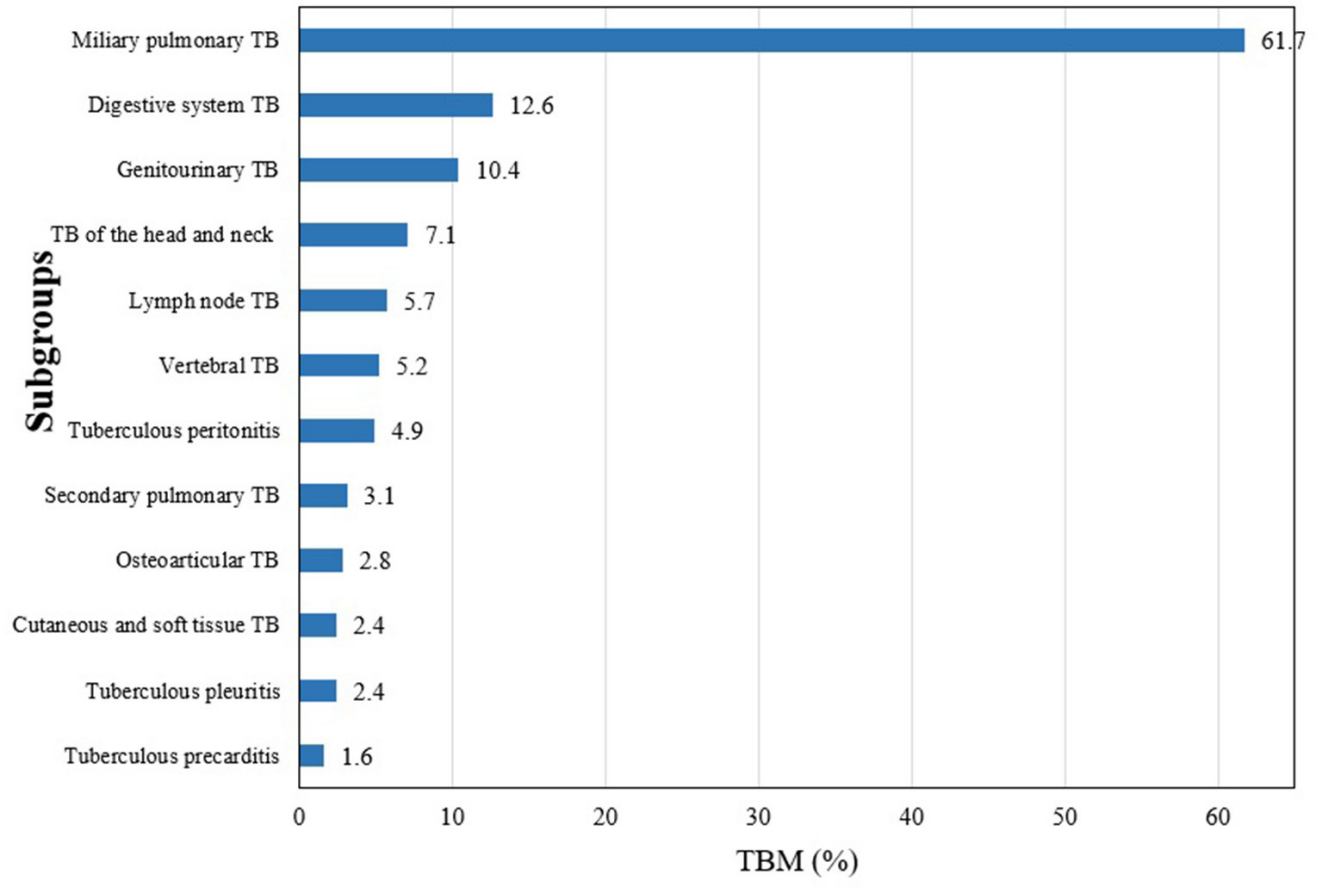
The proportion of TBM in different types of tuberculosis.

Among the 127 definite TBM cases, 4 (3.1%) of them were positive with smear microscopy, 23 (18.1%) for Mycobacteria Growth Indicator Tube (MGIT) culture, 24 (18.9%) for Lowenstein-Jenson culture, 42 (33.1%) for PCR, and 74 (58.3%) for Xpert, and two were pathologically diagnosed by biopsy from the brain (Figure 1). Additionally, although the hospitalized cases of TB increased gradually from 2,066 in 2012 to 3,475 in 2019, the proportion of TBM showed a decreasing trend from 4.4 to 2.4% during the same period (Figure 2). Miliary pulmonary TB (61.7%) with TBM was the highest, followed by digestive system TB (12.6%) and genitourinary TB (10.4%) (Figure 3).

When comparing patients with TB to patients without TBM, the univariate analysis showed that age, sex, marriage, occupation, comorbidities (including diabetes mellitus, hypertension, coronary heart disease, rheumatic disease, carcinoma, and malnutrition), and concurrent TB (miliary pulmonary TB, tuberculous pleuritis, lymph node TB, TB of the head and neck, digestive system TB, genitourinary TB, and vertebral TB) were significantly different among the three groups (*p* < 0.05), as reported in Table 1.

### Risk factors for definite TBM and probable TBM

Table 2 shows the results of the logistic regression model for factors related to definite TBM and probable TBM. In the present multivariable logistic regression model, compared with patients without TBM, patients aged < 30 years were associated with a greater likelihood of definite TBM compared with patients aged >59 years [aOR = 3.015, 95% CI: (1.451–6.266)]. Farmers were more likely to have definite TBM [aOR = 1.490, 95%CI: (1.020–2.177)]. Compared with patients without miliary pulmonary TB, patients with miliary pulmonary TB increased the risk of definite TBM [aOR = 105.842, 95%CI: (71.704–156.235)]. Malnutrition also increased the risk of definite TBM [aOR = 2.466, 95%CI: (1.110–5.479)]. Additionally, probable TBM was significantly associated with patients aged < 30 years [aOR = 2.174, 95% CI: (1.450–3.261)], aged 30–59 years [aOR = 1.670, 95%CI: (1.222–2.282)], who are farmers [aOR = 1.482, 95%CI: (1.203–1.825)], with miliary pulmonary TB [aOR = 108.696, 95%CI: (87.122–135.613)], and with digestive system TB [aOR = 2.906, 95%CI: (1.762–4.793)] (Table 2).

**Table 2:** Associated factors for definite TBM and probable TBM.

**Variables**	**Definite TBM (vs. non-TBM)**		**Probable TBM (vs. non TBM)**	
	**aOR (95% CI)**	**P value**	**aOR (95% CI)**	**P value**
**Age group, years**				
≥60	Ref		Ref	
< 30	3.015 (1.451–6.266)	**0.003**	2.174 (1.450–3.261)	**< 0.001**
30–59	1.348 (0.736–2.468)	0.333	1.670 (1.222–2.282)	**0.001**
**Occupation**				
Other	Ref		Ref	
Farmer	1.490 (1.020–2.177)	**0.039**	1.482 (1.203–1.825)	**< 0.001**
Malnutrition	2.466 (1.110–5.479)	**0.027**	1.423 (0.820–2.470)	0.210
Miliary pulmonary TB	105.842 (71.704–156.235)	**< 0.001**	108.696 (87.122–135.613)	**< 0.001**
Tuberculous pleuritis	0.563 (0.340–0.930)	**0.025**	0.672 (0.514–0.879)	**0.004**
Digestive system TB	0.945 (0.280–3.186)	0.927	2.906 (1.762–4.793)	**< 0.001**

TBM, tuberculous meningitis; TB, tuberculosis; aOR, adjusted odds ratio.

The value of *P* < 0.05 indicates statistical significance.

## Discussion

The present study found that 3.1% of TBM was prevalent among patients with TB, which was consistent with those given in previous studies (2, 3, 27). The present study also found that patients aged < 30 years, being farmers, and having miliary pulmonary TB were associated with a higher risk of definite and probable TBM. Additionally, malnutrition increased the probability of definite TBM, while digestive system TB increased the probability of probable TBM. Meanwhile, tuberculous pleuritis was associated with decreased probability.

Our study reported patients with TB who were < 30 years of age were two to three times more likely to have TBM, and compared with patients aged > 59 years, younger patients were more susceptible to TBM. Previous studies observed that TBM affected young children most commonly, and the peak was 2–4 years old, while the progression from latent tuberculosis infection (LTBI) to more severe forms of TB disease was faster in younger children (~1–4 months for TBM) (28–30). However, there is no report about patients aged < 30 years who are more prone to suffer from TBM. A retrospective cohort study from China found that patients aged > 20 years had a significantly lower risk for treatment delay (31). The possible reason was that younger patients are more likely to come to the hospital to seek health care, once they have suspected symptoms of TB. Additionally, the present study also observed that farmer was associated with an increased probability of TBM, while the results on farmers had not been reported in previous studies. Wang et al. reported that the farmer increased the risk of TB (14). Possible reasons were that farmers have a relatively low income, live in a relatively crowded poor environment, and are malnourished, thus making them vulnerable to TB. Second, farmers have low education level and poor knowledge of TB, which are the other reasons why they do not seek medical care when they are suspected symptoms of TB (32–34). However, TB progresses without treatment, which increases the probability of TBM. Therefore, increasing income and improving living conditions are helpful to prevent TBM from affecting farmers. Moreover, an appropriate way of promoting health education is to propagate the knowledge of TB among farmers, especially among illiterate farmers, which in turn promotes them to seek timely healthcare, which plays a more important role in reducing the incidence of TBM.

Miliary pulmonary TB results from the hematogenous spread of mycobacterium tuberculosis (Mtb) in the pulmonary. The present study found that 61.7% of patients with miliary pulmonary TB had TBM, which was similar to those mentioned in previous studies (11, 21). On the other hand, more than one-half (56.6%) of the patients with TBM had miliary pulmonary TB and only 1.1% of patients without TBM had miliary pulmonary TB. Miliary pulmonary TB was the most common hematogenous TB, followed by digestive system TB (30). In the present study, patients with miliary pulmonary TB had more than 100 times increased risk of both definite and probable TBM compared with TB patients without miliary pulmonary TB, and digestive system TB was also associated with increased risk for probable TBM. Lymph node TB, vertebral TB, and TB of the head and the neck were not associated with the risk for TBM, and the results indicated from the TBM pathogenesis mentioned previously that TBM was secondary to hematogenous spread, other than lymphangitic or contiguous spread. The results also suggested that, when physicians encounter such patients they consider performing cerebral imaging and lumbar puncture to exclude TBM for TB patients with miliary pulmonary TB in clinical practice.

The results of the present study observed that malnutrition increased the risk of definite TBM. The cellular immunity plays a critical role in immune responses to Mtb infection. Malnutrition could impair T-cell function, particularly the production of T-helper-1 cytokines and functions of the macrophage antimycobacterial effector (35). A previous study demonstrated that malnutrition increased the incidence and exacerbated clinical manifestations of TB (36). Patients with TB have an increased metabolism and a decreased appetite that compounds the already present malnutrition. Thus, a merciless vicious circle between TB and malnutrition continues (37). Previous studies revealed that malnutrition increased the risk of death for TB (38) and TBM (30). Improving the living conditions and reducing malnutrition can not only reduce the occurrence of TB and TBM but also improve the prognosis.

Our results found that tuberculous pleuritis was related to decreased risk for definite and probable TBM. The reason can be given that most patients with tuberculous pleuritis have fever, chest pain, shortness of breath, etc. Urgently, these types of discomfort promote patients to seek healthcare, while the diagnosis and treatment of tuberculous pleuritis reduce its progression to TBM. In addition, tuberculous pleuritis decreases the probability of TBM that may relate to their pathogenesis, but the pathogenesis of these two diseases is not so clear; hence, further studies need to be conducted in the future.

There are some limitations to our study. First, the study included inpatients only from a single center in China. Second, patients with HIV infection had not been analyzed further based on a small sample with 23 cases. In addition, the present study did not collect information about smoking and drinking, which is likely to correlate with TBM.

In summary, the present data reported on the proportion of TBM, including definite and probable TBM among patients with TB from a large sample study, and revealed risk factors including patients aged < 30 years, being farmers, and having miliary pulmonary TB affecting patients with TBM. The results of the present study suggested screening patients with TB having the aforementioned characteristics that could facilitate clinicians to identify patients with TBM at an early stage and improve the prognosis of patients with TBM further. Further studies should be conducted to confirm the findings of the present study.

## Data availability statement

The original contributions presented in the study are included in the article, further inquiries can be directed to the corresponding authors.

## Ethics statement

The study was approved by the Ethics Committee of Beijing Chest Hospital. In our study, the retrospective collection and analysis of case were patients' demographic characteristics and diagnosis information. All data were supplied and analyzed, without access to personal identifying information. No informed consent was required.

## Author contributions

MH, YM, QL, and NC were involved in the conception and design of the project. MH and YM carried out the analysis and wrote the first draft of the manuscript. MH, YM, XJ, HJ, and FL conducted the literature search, data acquisition, and input data. QL had full access to all the data in the study and had final responsibility for the decision to submit for publication. All authors read and contributed to the final manuscript.

## References

[1] World Health Organization. Global tuberculosis report 2022. Geneva. Available online at: https://www.who.int/teams/global-tuberculosis-programme/tb-reports/global-tuberculosis-report-2022

[2] Torok ME. Tuberculous meningitis: advances in diagnosis and treatment. Brit Med Bull. (2015) 113:117–31. 10.1093/bmb/ldv003 25693576

[3] Lincoln EM. Tuberculous meningitis in children; with special reference to serous meningitis; tuberculous meningitis. Am Rev Tuberc. (1947) 56:75–94. 10.1164/art.1947.56.2.75 20264373

[4] Seddon JA Wilkinson R van Crevel R Figaji A Thwaites GE Tuberculous MIRC. Knowledge gaps and research priorities in tuberculous meningitis. Wellcome Open Res. (2019) 4:188. 10.12688/wellcomeopenres.15573.1 32118120 PMC7014926

[5] Thao LTP Heemskerk AD Geskus RB Mai NTH Ha DTM Chau TTH. Prognostic models for 9-month mortality in tuberculous meningitis. Clin Infect Dis. (2018) 66:523–32. 10.1093/cid/cix849 29029055 PMC5850565

[6] Wen L Li M Xu T Yu X Wang L Li K. Clinical features, outcomes and prognostic factors of tuberculous meningitis in adults worldwide: systematic review and meta-analysis. J Neurol. (2019) 266:3009–21. 10.1007/s00415-019-09523-6 31485723

[7] Wang M Luo L Zhang Y Liu X Liu L He J. Treatment outcomes of tuberculous meningitis in adults: a systematic review and meta-analysis. BMC Pulm Med. (2019) 19:1. 10.1186/s12890-019-0966-8 31694599 PMC6833188

[8] Thwaites GE Duc Bang N Huy Dung N Thi Quy H Thi Tuong Oanh D Thi Cam Thoa N. The influence of HIV infection on clinical presentation, response to treatment, and outcome in adults with tuberculous meningitis. (2005) 192:2134–41. 10.1086/498220 16288379

[9] Heemskerk AD Bang ND Mai NTH Chau TTH Chau NVV Phu NH. Intensified antituberculosis therapy in adults with tuberculous meningitis. N Engl J Med. (2016) 374:124–34. 10.1056/NEJMoa1507062 26760084

[10] Bahr NC Boulware DR. Methods of rapid diagnosis for the etiology of meningitis in adults. Biomark Med. (2014) 8:1085–103. 10.2217/bmm.14.67 25402579 PMC4239990

[11] Venkatraman N King T Bell D Woltmann G Wiselka M Abubakar I. High levels of neurological involvement but low mortality in miliary tuberculosis: a 6-year case-series from the UK. Eur Respir J. (2016) 47:1578–81. 10.1183/13993003.01958-2015 26846825

[12] Li XL Xie N Wang SW Wu QH Ma Y Shu W. Diagnostic value of cerebrospinal fluid T-SPOTTB for tuberculousis meningitis in China. Biomed Environ Sci. (2017) 30:681–4. 10.3967/bes2017.091 29081344

[13] Gupta BK Bharat A Debapriya B Baruah H. Adenosine deaminase levels in CSF of tuberculous meningitis patients. J Clin Med Res. (2010) 2:220–4. 10.4021/jocmr429w 21629544 PMC3104661

[14] Wang X Yin S Li Y Wang W Du M Guo W. Spatiotemporal epidemiology of, and factors associated with, the tuberculosis prevalence in northern China, 2010-2014. BMC Infect Dis. (2019) 19:365. 10.1186/s12879-019-3910-x 31039734 PMC6492399

[15] Dooley KE Chaisson RE. Tuberculosis and diabetes mellitus: convergence of two epidemics. Lancet Infect Dis. (2009) 9:737–46. 10.1016/S1473-3099(09)70282-8 19926034 PMC2945809

[16] Shu C Wei Y Yeh Y Lin H Chen C Wang P. The impact on incident tuberculosis by kidney function impairment status: analysis of severity relationship. Resp Res. (2020) 21:51. 10.1186/s12931-020-1294-5 32050967 PMC7017479

[17] Cho PJ Wu C Johnston J Wu M Shu C Lin H. Progression of chronic kidney disease and the risk of tuberculosis: an observational cohort study. Int J Tuberc Lung Dis. (2019) 23:555–62. 10.5588/ijtld.18.0225 31097063

[18] Vuorela M Mars NJ Salonen J Kauppi MJ. Tuberculosis in people with rheumatic disease in Finland 1995-2007: a nationwide retrospective register study. Rheumatol Adv Pract. (2019) 3:z20. 10.1093/rap/rkz020 31528842 PMC6736076

[19] Davis AG Rohlwink UK Proust A Figaji AA Wilkinson RJ. The pathogenesis of tuberculous meningitis. J Leukocyte Biol. (2019) 105:267–80. 10.1002/JLB.MR0318-102R 30645042 PMC6355360

[20] Donald P Schaaf H Schoeman J. Tuberculous meningitis and miliary tuberculosis: the Rich focus revisited. J Infection. (2005) 50:193–5. 10.1016/j.jinf.2004.02.010 15780412

[21] Garg RK Sharma R Kar AM Kushwaha RAS Singh MK Shukla R. Neurological complications of miliary tuberculosis. Clin Neurol Neurosur. (2010) 112:188–92. 10.1016/j.clineuro.2009.11.013 20031301

[22] Thwaites GED van Toorn RM Schoeman JP. Tuberculous meningitis: more questions, still too few answers. Lancet Neurology. (2013) 12:999–1010. 10.1016/S1474-4422(13)70168-6 23972913

[23] Marais S Thwaites G Schoeman JF Torok ME Misra UK Prasad K. Tuberculous meningitis: a uniform case definition for use in clinical research. Lancet Infect Dis. (2010) 10:803–12. 10.1016/S1473-3099(10)70138-9 20822958

[24] Hussain SF Irfan M Abbasi M Anwer SS Davidson S Haqqee R. Clinical characteristics of 110 miliary tuberculosis patients from a low HIV prevalence country. Int J Tuberc Lung Dis. (2004) 8:493 15141744

[25] Sharma SK Mohan A Sharma A Mitra DK. Miliary tuberculosis: new insights into an old disease. Lancet Infect Dis. (2005) 5:415–30. 10.1016/S1473-3099(05)70163-8 15978528

[26] Cederholm T Bosaeus I Barazzoni R Bauer J Van Gossum A Klek S. Diagnostic criteria for malnutrition - an ESPEN consensus statement. Clini Nutr. (Edinburgh, Scotland). (2015) 34:335–40. 10.1016/j.clnu.2015.03.001 25799486

[27] Seddon JA Tugume L Solomons R Prasad K Bahr NC. Tuberculous MIRC. The current global situation for tuberculous meningitis: epidemiology, diagnostics, treatment and outcomes. Wellcome Open Res. (2019) 4:167. 10.12688/wellcomeopenres.15535.1 32118118 PMC7029758

[28] Duque-Silva A Robsky K Flood J Barry PM. Risk factors for central nervous system tuberculosis. Pediatrics. (2015) 136:e1276–84. 10.1542/peds.2014-3958 26438712

[29] Donovan J Thwaites GE Huynh J. Tuberculous meningitis: where to from here? Curr Opin Infect Dis. (2020) 33:259–66. 10.1097/QCO.0000000000000648 32324614 PMC7259381

[30] Starke JR. Tuberculosis in children. Semin Resp Crit Care. (2004) 25:353–64. 10.1055/s-2004-829507 16088476

[31] He Y Han C Chang K Wang M Huang T. Total delay in treatment among tuberculous meningitis patients in China: a retrospective cohort study. BMC Infect Dis. (2017) 17:341. 10.1186/s12879-017-2447-0 28499348 PMC5429562

[32] Asres M Gedefaw M Kahsay A Weldu Y. Patients' delay in seeking health care for tuberculosis diagnosis in East Gojjam zone, Northwest Ethiopia. Am J Trop Med Hyg. (2017) 96:1071–5. 10.4269/ajtmh.16-0892 28500803 PMC5417197

[33] Luba TR Tang S Liu Q Gebremedhin SA Kisasi MD Feng Z. Knowledge, attitude and associated factors towards tuberculosis in Lesotho: a population based study. BMC Infect Dis. (2019) 19:96. 10.1186/s12879-019-3688-x 30696417 PMC6352435

[34] Ali Warsi SM Danish SH Ahmad F Khan AI Khan MP Bano S. Tuberculosis knowledge and health seeking behaviour: a tale of two districts of Sindh, Pakistan. JPMA. (2016) 66:1120–6 27654732

[35] Boelaert JR Gordeuk VR. Protein energy malnutrition and risk of tuberculosis infection. Lancet. (2002) 360:1102. 10.1016/S0140-6736(02)11169-X 12384022

[36] Macallan DC. Malnutrition in tuberculosis. Diagn Micr Infec Dis. (1999) 34:153–7. 10.1016/s0732-8893(99)00007-3 10354866

[37] Martin SJ Sabina EP. Malnutrition and associated disorders in tuberculosis and its therapy. J Diet Suppl. (2019) 16:602–10. 10.1080/19390211.2018.1472165 29958051

[38] Zachariah R Spielmann MP Harries AD Salaniponi FML. Moderate to severe malnutrition in patients with tuberculosis is a risk factor associated with early death. T Roy Soc Trop Med H. (2002) 96:291–4. 10.1016/s0035-9203(02)90103-3 12174782

